# Phylogeny and phylogeography of functional genes shared among seven terrestrial subsurface metagenomes reveal N-cycling and microbial evolutionary relationships

**DOI:** 10.3389/fmicb.2014.00531

**Published:** 2014-10-31

**Authors:** Maggie C. Y. Lau, Connor Cameron, Cara Magnabosco, C. Titus Brown, Faye Schilkey, Sharon Grim, Sarah Hendrickson, Michael Pullin, Barbara Sherwood Lollar, Esta van Heerden, Thomas L. Kieft, Tullis C. Onstott

**Affiliations:** ^1^Department of Geosciences, Princeton UniversityPrinceton, NJ, USA; ^2^National Center for Genome ResourcesSanta Fe, NM, USA; ^3^Department of Computer Science and Engineering and Department of Microbiology and Molecular Genetics, Michigan State UniversityEast Lansing, MI, USA; ^4^The Marine Biological LaboratoryWoods Hole, MA, USA; ^5^Department of Chemistry, New Mexico TechSocorro, NM, USA; ^6^Department of Earth Sciences, University of TorontoToronto, ON, Canada; ^7^Department of Biotechnology, University of Free StateBloemfontein, South Africa; ^8^Department of Biology, New Mexico TechSocorro, NM, USA

**Keywords:** terrestrial subsurface, functional genes, phylogeography, phylogeny, phylogenetics, N-cycle, evolution

## Abstract

Comparative studies on community phylogenetics and phylogeography of microorganisms living in extreme environments are rare. Terrestrial subsurface habitats are valuable for studying microbial biogeographical patterns due to their isolation and the restricted dispersal mechanisms. Since the taxonomic identity of a microorganism does not always correspond well with its functional role in a particular community, the use of taxonomic assignments or patterns may give limited inference on how microbial functions are affected by historical, geographical and environmental factors. With seven metagenomic libraries generated from fracture water samples collected from five South African mines, this study was carried out to (1) screen for ubiquitous functions or pathways of biogeochemical cycling of CH_4_, S, and N; (2) to characterize the biodiversity represented by the common functional genes; (3) to investigate the subsurface biogeography as revealed by this subset of genes; and (4) to explore the possibility of using metagenomic data for evolutionary study. The ubiquitous functional genes are *Nar*V, NPD, PAPS reductase, *Nif*H, *Nif*D, *Nif*K, *Nif*E, and *Nif*N genes. Although these eight common functional genes were taxonomically and phylogenetically diverse and distinct from each other, the dissimilarity between samples did not correlate strongly with geographical or environmental parameters or residence time of the water. *Por* genes homologous to those of *Thermodesulfovibrio yellowstonii* detected in all metagenomes were deep lineages of Nitrospirae, suggesting that subsurface habitats have preserved ancestral genetic signatures that inform the study of the origin and evolution of prokaryotes.

## Introduction

Solar energy and photosynthesis together form the basis for life to thrive in most ecosystems on Earth, except where temperature is too hot for the photosynthetic machinery to operate (e.g., geothermal springs Lau et al., 2006), or where it is too deep for light or photosynthetically-derived carbon substrates to penetrate (e.g., terrestrial deep subsurface environments Lin et al., 2006). In the terrestrial deep subsurface, chemical energy sources such as H_2_, CH_4_, SO^2−^_4_ and hydrocarbons are generated by radiolysis, thermogenesis, water-rock interactions or microbial activity (Kieft et al., 2005; Lin et al., 2006; Onstott et al., 2006; Etiope and Sherwood Lollar, 2013). The reliance on chemical energy sources to fuel primary production in these reducing environments has made the deep subsurface biome an analog for investigations of ancient microbial life in the Archean (4-2.5 billion years ago) and of potential extraterrestrial subsurface habitats (Gold, 1992). Surveys of microbial community structure in deep subsurface sites have been carried out on different continents (Pedersen, 1997; Zhang et al., 2005; Christner et al., 2006; Gihring et al., 2006; Sahl et al., 2008; Fry et al., 2009; Itävaara et al., 2011; Dong et al., 2014), however, little is known about the factors governing their distribution patterns over spatial and temporal scales.

Surface microbial communities display distribution patterns over large spatial scales (>10^3^ km) as a result of geographical and/or environmental characteristics (see review in Martiny et al., 2006). Two seminal papers challenged the dogma of “everything is everywhere, but the environment selects” (Baas Becking, 1934) by showing that the geographic distance of separation better explains the degree of genetic variation among hot spring inhabitants, specifically the cyanobacterium *Synechococcus* spp. (Papke et al., 2003) and the archaeon *Sulfolobus* spp. (Whitaker et al., 2003). The interplay of geographical isolation, microbial dispersal and subsequent selection has defined the microbial biogeography in these extreme ecosystems.

Evaluations of microbial biogeography of surface habitats has been drawn mainly from taxonomic data derived from 16S ribosomal RNA (rRNA) genes using various culture-independent approaches (e.g., PCR-cloning, TRFLP, pyrosequencing, and metagenomics). Depending on the habitat and spatial scale studied, the microbial diversity may be governed by environmental factors, such as pH (Lauber et al., 2009), soil moisture content (Angel et al., 2010), or physical locality (Lau et al., 2009; Schmidt et al., 2011).

Unfortunately, taxonomic identity of a microorganism does not always correspond well with its functional role in a particular community, owing to (1) the physiological promiscuity encoded within its genome (Medini et al., 2005), (2) differential gene expression under different conditions (Hottes et al., 2004), and (3) acquisition of exotic genes from a species of different genus or higher taxonomic rank via horizontal gene transfer (HGT) or phage-mediated translocation (Chivian et al., 2008; Kunisawa, 2010). It has been shown that the dominant forces shaping taxonomic vs. functional compositions are not the same within a gene family (Beier et al., 2011) or within a community (Raes et al., 2011). Hence, the use of taxonomic assignments or patterns may give limited inference on how microbial functions are affected by historical, geographical and environmental factors. Functional traits that affect cell fitness therefore have more direct relevance (Green et al., 2008) and metagenomic data is largely comprised of protein-coding genes (Raes et al., 2011).

Metagenomes of oceanic samples have recently been exploited to investigate the pattern of functional traits in relation to geographical distance and environmental parameters. Using unassembled metagenomic data from the Global Ocean Survey, two studies (Raes et al., 2011; Jiang et al., 2012b) concluded that differences in functional traits of surface ocean communities correlate more strongly with environmental factors than with physical distance, even though the next-generation sequencing reads were annotated using different databases (KEGG vs. Pfam) and different dimensional reduction methods were employed (canonical correlation analysis vs. non-negative matrix factorization).

It has been postulated that the terrestrial deep biosphere is volumetrically greater than the surface and marine biospheres combined, and it has been estimated to account for more than 40–50% of the global biomass, thus containing an enormous genetic capacity (Whitman et al., 1998). In many respects the fluid-filled fractures in the deep continental fractured rock environments are similar to surface hot springs because they provide the greatest opportunity for nutrient acquisition and mobility compared to the surrounding rock matrix that has low porosity, low permeability and often low water availability. However, unlike hot spring environments, microbial dispersal through wind (one of the global dispersal vectors) does not directly affect the microbial biogeography of these deep, isolated continental oases. The access of windborne microorganisms to these deep terrestrial subsurface environments can occur only indirectly by infiltration through pore spaces with precipitation to the water table, followed by transport with groundwater flow.

In shallower aquifers, microbial migration over a distance of 0.6 km through a highly porous (35%) sandy aquifer, with a ground water velocity of <1 m day^−1^, was affected by cell size (Harvey and Garabedlan, 1991) and other cellular characteristics (Mailloux et al., 2003). However, the cell concentration of the migrating bacterial species decreased by an order of magnitude over a horizontal distance of only 7 m (Mailloux et al., 2003). This means that any recharge of surface microorganisms is highly attenuated before reaching even 100 m depth, and some adaptation combined with growth is required for subsurface microbial dispersion over long distances, even in high permeability porous media. Geological tests have shown that microorganisms traveled through the less porous sandstones (8.5–20%) at Cerro Negro, New Mexico, USA over a distance of 100 m, at depth of ~300 m, in less than 3.4 million years via a ground water velocity of 0.1 m yr^−1^ (Walvoord et al., 1999). The microbial colonization of a low permeability sterilized sandstone (porosity of 1–12%) in the Piceance Basin, western Colorado, USA down to the depth of 860 m had occurred in less than 5 million years (Colwell et al., 1999). The microbial transport in fractured basement rocks is even less certain, as these rocks typically exhibit a bimodal hydraulic conductivity/porosity with fractures yielding water velocity of ~3 cm yr^−1^, but comprising only 0.01% of the porosity, and a matrix porosity of ~1% yielding water velocity of ~0.003 cm yr^−1^ (Nordstrom et al., 1989). Although the transport of microbial communities in the deep terrestrial subsurface on the 100-km spatial scale and million-year time scale is poorly constrained, it is hypothesized that this apparently restricted connectivity between fractures influences subsurface genetic exchange and results in the divergence of subsurface microbial genomes from their surface counterparts through genetic drift. Therefore, the deep terrestrial subsurface is a high priority target for the study of microbial biogeography that has received little attention until this study.

Unfortunately, the molecular data on metabolic functions is still too little to inform what functions or pathways of biogeochemical cycling of CH_4_, S, and N would be ubiquitous in terrestrial deep subsurface habitats, let alone their geographic distribution. Not until the diversity of functional traits and their phylogeographical patterns being resolved, we can only speculate on what the driving forces are for functional phylogeography in the terrestrial deep subsurface. To date, the main restriction to addressing these questions has been limited accessibility to subsurface samples with little contamination over space and time to perform comparative analyses of their microbial communities.

Deep mines and underground laboratories, however, do provide a relatively inexpensive means of sampling deep groundwater at multiple points in time and space. With the use of established sampling techniques to minimize potential contaminants, planktonic microbial biomass were recently collected from seven fracture water samples that were >1.1 km depth in five mines in South Africa. The objectives of this study were to undertake a combined taxonomic- and phylogeny-based approach (1) to describe the distribution of the functional genes encoding CH_4_, S, and N metabolism that were shared among all seven metagenomes; (2) to investigate the relatedness of these metagenomes; (3) to examine their correlation to geographic distance, environmental parameters and groundwater residence time; and (4) to explore the possibility of utilizing metagenomic data for evolutionary study.

## Materials and methods

### Field sites

Four of our study sites are located in the Witwatersrand Basin, which is located in the center of the Kaapvaal Craton of South Africa. The sedimentary and volcanic strata of the Witwatersrand Basin, deposited between 2.9 and 2.5 billion years ago, were intruded along the northern margin by the 2.05 Ga Bushveld Igneous Complex and subjected to a meteorite impact 2.0 billion years ago that led to the formation of the Vredefort dome that currently sits at the center of the 300 km long along a NE-SW axis and 100 km wide basin (Frimmel, 2005). Because of uplift and erosion after 90 Ma the subsurface sites cooled to their current temperatures 30 million years ago (Omar et al., 2003). Driefontein (DR) and Tau Tona (TT) Au mines, 7.8 km apart, are situated near the northwestern edge of the basin. Beatrix (BE) and Masimong (MM) Au mines, 28 km apart, are situated near the southwestern margin of the basin. They are ~170–200 km southwest of DR and TT. Finsch (FI) diamond mine is the fifth study site and is on the Ghaap Plateau 370 km west of BE Au mines.

Two samples, BE326FW250111 Bh2 (BE2011) and BE326FW270712 Bh2 (BE2012), were collected in 2011 and 2012, respectively, from borehole BE326 (shaft #3 level 26). It is located at a depth of 1.34 km (Table 1). This sub-horizontal borehole penetrates 57 m into a medium to coarse-grained sub-lithic arenite to intersect a NNW striking fault zone where it encountered high-pressure water with a flow rate of 750 L min^−1^. Since it was first drilled in 2007, it has been sealed off with a high-pressure steel valve. From this same borehole, a novel subsurface nematode *Halicephalobus mephisto* was isolated (Borgonie et al., 2011).

**Table 1:** **Geographical, physical, and chemical characteristics of the boreholes**.

**Sample code**	**Sample ID**	**Latitude**	**Longitude**	**Mine**	**District**	**Depth (mbls)**	**Residence time (kyr)^a^**	**δ^18^O(‰)**	**δ^2^H(‰)**
BE2011	BE326FW250111 Bh2	S 28°14′24″	E 26°47′45″	Beatrix	Welkom	1339	>40–80	−5.94	−40.98
BE2012	BE326FW270712 Bh2	S 28°14′24″	E 26°47′45″	Beatrix	Welkom	1339	>40–80	−8.68	−47.00
DR5	DR5IPCFW280711	S 26°26′05″	E 27°30′14″	Driefontein	Carletonville	1046	16–24	−4.32	−24.56
FI88	FI88FW031012	S 28°22′42″	E 23°26′45″	Finsch	Finsch	1056	~410	−6.17	−38.30
MM5	MM5.1940(46)FW200712	S 27°58′52″	E 26°52′30″	Masimong	Welkom	1900	>BE	−7.00	−40.00
TT107	TT107FW240811	S 26°25′05″	E 27°25′38″	Tau Tona	Carletonville	3048	1–6	−5.11	−22.40
TT109	TT109FW060312 Bh2	S 26°25′05″	E 27°25′38″	Tau Tona	Carletonville	3136	16–21	−5.02	−25.29

**Table:** 

**Sample code**	***T* °C**	**pH**	**DOC μM**	**DIC mM**	**TN μM**	**NO^-^_2_ μM**	**NO^-^_3_ μM**	**NH^4+^ μM**	**N_2_ mM**	**H_2_ mM**	**CH_4_ mM**	**Na^+^ mM**	**K^+^ mM**	**Mg^2+^ μM**	**Ca^2+^ mM**	**Sr^2+^ μM**	**Ba^2+^ μM**	**Si^4+^ μM**	**Mn^2+^ μM**	**Fe^2+^ μM**	**F^-^ μM**	**Cl^-^ mM**	**Br^-^ mM**	**SO^2-^_4_ μM**
BE2011	36.9	8.8	16.3	0.5	29.5	3.9	0.4	83.2	0.4	0.13	2.0	78.0	0.7	0.06	2.87	8.0	9.7	0.4	0.7	< d.l.	0.1	69.8	0.2	0.1
BE2012	38.1	8.6	28.8	-	47.1	< d.l.	6.0	46.6	0.4	0.009	1.0	48.2	0.9	0.03	3.88	73.4	10.1	0.4	5.6	0.6	0.1	61.5	0.1	0.6
DR5	26.8	7.4	85	2.4	3.0	0.1	14.7	1.9	0.5	0.003	0.03	2.2	0.1	0.4	0.50	5.6	0.5	0.3	0.2	0.6	0.2	1.4	0.003	0.1
FI88	28.9	6.8	130	0.05	-	1.8	15.1	-	-	-	-	9.9	0.2	0.2	5.61	10.3	0.3	0.4	5.0	0.4	0.02	21.5	0.03	0.3
MM5	40.7	7.7	45	0.4	32.1	< d.l.	1.0	-	1.4	0.19	8.9	45.1	0.4	0.1	1.89	30.5	6.6	0.3	6.0	0.9	0.1	55.0	0.2	0.01
TT107	52.1	8.6	18.3	0.6	3.2	< d.l.	1.0	3.9	6.0	17.13	8.8	2.5	0.03	0.04	0.27	3.2	0.6	0.5	0.1	0.1	0.1	2.8	0.01	0.1
TT109	48.7	8.2	39.2	0.7	5.5	< d.l.	0.1	2.2	0.9	0.35	2.3	3.4	0.04	0.03	0.52	3.1	0.5	0.5	-	0.6	0.1	3.2	0.01	0.1

Abbreviations: DOC, dissolved organic carbon; DIC, dissolved inorganic carbon; TN, total nitrogen; <d.l., below detection limit; and “-,” missing data.

a Age of fracture water from FI was determined by ^81^Kr dating carried out in this study; otherwise, it was determined by radiocarbon dating of DIC (Simkus et al., in preparation) and noble gases (Lippmann et al., 2003).

Two samples were collected from different boreholes at TT Au mine, namely TT107FW240811 from level 107 (TT107) and TT109FW060312 Bh2 from level 109 (TT109) (Table 1). TT107 is a sub-horizontal borehole located at 3.05 km depth and penetrates 400 m into medium-grained quartzite, crossing the 100 m wide Pretorius Fault Zone (Heesakkers et al., 2011) and intersecting the border of the NNE striking Jeans Dyke. TT109 is a sub-horizontal borehole located at 3.14 km depth and penetrates 100 m into medium-grained quartzite to also intersect the border of Jeans Dyke. The water intersections of TT107 and TT109 are separated by ~100 m horizontally as well as ~100 m vertically. Both of these boreholes were sealed off after intersecting water with high-pressure steel valves just several weeks prior to collecting the samples.

Sample DR5IPCFW280711 (DR5) was collected from a valved horizontal borehole very close to the DR Au mine shaft #5 at a depth of 1.05 km (Table 1). This is an old borehole that penetrates the Malmani Subgroup dolomite aquifer of the 2.45 Ga Transvaal Supergroup and was designed to tap the fracture water but never used. At this location the dolomite is completely overlain by banded iron formation that has confined the water flow occurring primarily through fractures in the dolomite. This borehole is located 3.8 km from the water intersections of TT107 and TT109.

Sample MM51940(46)FW200712 (MM5) was collected from the MM Au mine shaft #5 level 46 at a depth of 1.90 km (Table 1). The borehole penetrates quartzite and intersects the water-bearing Saaiplaas Fault. The borehole was tilted ~45° upwards and sealed with a high pressure valve.

Sample FI88FW031012 (FI88) was collected from FI diamond mine level 88 at a depth of 1.06 km (Table 1). This vertical borehole penetrates 175 m of Transvaal Supergroup age Ghaap Plateau dolomites, where it intersects an artesian fracture, and was left as an open flowing borehole with water flow rate of 20 L min^−1^.

### Sampling

At each site, a sterile stainless steel manifold, with all 7 valves fully open, was connected to the borehole casing as a means of excluding mine air and other contaminants. The main valve was opened to let the fracture water that was under natural high pressure to gush out for several minutes. This flushed out water that might have been oxygenated during the initial contact with mine air and also flushed air out of the sterile manifold. In the case of FI88, a sterile 1-m long Margot-type packer was inserted into the borehole with the manifold attached directly to it. Sterile sampling tubes subsequently connected to the manifold were flushed in a similar manner immediately after installation. The openings of the 7 valves on the manifold were adjusted in order to accommodate the collection of various sample types at desired water flow rates. A pre-autoclaved set of a pleated Memtrex NY filter (Cat. No. MNY-91-1-AAS or MNY-92-1-AAS, General Electric Co.) housed in a 25-cm long stainless steel filter holder was connected to the manifold with a water flow rate set at 4 L min^−1^. The filter was recovered after days or weeks, depending on mining operations, thus the filtration periods differed.

During the retrieval trip, the filter holder was disconnected, the water inside was decanted through the inlet or outlet, then refilled with sterile RNA-preservation solution and sealed with sterile threaded plugs. The solution contained 20 mM Ethylenediaminetetraacetic acid (EDTA), 0.3 M sodium citrate and 4.3 M ammonium sulfate; the pH was adjusted to 5.2 using concentrated H_2_SO_4_. The formula of this super-saturated salt solution was based on Brown and Smith (2009) and Dr. Derek Jamieson's recipe (pers. comm.). The filter holder and the filter were put in a cooler of reusable ice packs on site or immediately upon reaching the surface. The filter was kept in the RNA-preservation solution at 4°C overnight to saturate all membrane layers. The filter was then aseptically transferred into double Ziploc^®^ bags (sterile from the manufacturer) and stored at −80°C until processing. A dry-shipper (model MVE XC20/3) was used to transport the filters and temperature-sensitive samples to the United States at continuous liquid N_2_ temperature.

Filtered water samples for anion and cation measurements were collected in Nalgene bottles following the methods described in Moser et al. (2003). A gas stripper was connected to the manifold for gas sampling. Dissolved gases were then transferred into pre-evacuated 160 mL vials using a 50 mL gas-tight syringe following the procedure described in Ward et al. (2004). For ^81^Kr analysis, the gas sample was collected from the fracture water using a leak-tight gas extraction system (Purtschert et al., 2013) and Kr purification was carried out by the Climate and Environmental Physics Department, University of Bern.

### Physical-chemical characterization

Basic water chemistry was measured at each site using CHEMET kits (Chemetrics, Inc., Calverton, VA), which included dissolved O_2_, Fe^2+^, total Fe, H_2_S, PO^3−^_4_, and H_2_O_2_. Temperature, pH and redox potential were measured using respective handheld probes (HANNA instruments, Woonsocket, RI).

Gas composition was determined for O_2_ and N_2_ (thermal conductivity detector), H_2_ and CO (reduced gas detector), and CO_2_ and CH_4_ (flame ionization detector) by gas chromatography (Peak Performer 1 series, Peak Laboratories, USA) using Ultra-High Purity (UHP) Ar as carrier gas. Sample dilution to instrumental linear response range was performed using UHP Ar. The anion concentrations were measured by an ion chromatograph coupled to an ESI-quadruple mass spectrometer (Dionex IC25 and Thermo Scientific MSQ, USA). The cation concentrations were determined by inductively-coupled-plasma optical emission spectroscopy, ICP-OES (Perkin Elmer Optima 4300 DV, USA). The NH^+^_4_ concentrations were determined by the phenol/hypochlorite method (Parsons et al., 1984). Dissolved inorganic carbon (DIC) was measured on an Aurora 1030W TOC Analyzer (OI Analytical, USA).

Total nitrogen (TN) was measured simultaneously with dissolved organic carbon (DOC) using a Shimadzu TOC-VCSH carbon analyzer with a TNM-L nitrogen analyzer. DIC was eliminated by acidification and sparging. The DOC was combusted and measured by the non-dispersive infrared sensor (NDIR) while the TN was measured by a chemiluminescence detector connected in series with the NDIR. The hydrogen and oxygen isotopic analyses of waters were performed at the Environmental Isotope Laboratory, University of Waterloo, Waterloo, Canada, following the procedure of Ward et al. (2004).

The concentrations of dissolved gases were derived from the gas volume abundance, the ratio of water to gas flow rates and Henry's law constants following the procedure of Andrews and Wilson (1987). These gas concentrations are considered minimum estimates because of the potential degassing of fluid internally within a partially depressurized fracture zone (Lippmann et al., 2003). Mineral solubility, charge balance, dissolved species activity, partial pressures and free energy of relevant reactions were calculated using the geochemical modeling program, The Geochemist's Workbench version 8.0 (Bethke, 2008).

Radiocarbon analyses of the DIC were performed by National Ocean Sciences Accelerator Mass Spectrometry (NOSAMS) facility at Woods Hole, MA, USA. Water samples were collected in 500 mL glass bottles with ground glass stoppers provided by NOSAMS. The samples were collected using the degassed manifold and tubing to overfill the bottle and were sealed immediately thereafter to ensure that the sample was not contaminated by air CO_2_. The residence time of fracture water from FI was determined by measuring the isotopic abundance of radionuclide ^81^Kr, which has a half-life of 229 kyr. The ^81^Kr/Kr was measured using the Atom Trap Trace Analysis system (Jiang et al., 2012a) at the Laboratory for Radiokrypton Dating at Argonne National Laboratory. The mean ^81^Kr groundwater residence time was calculated using the exponential law for radioactive decay.

### DNA extraction and purification

The MNY filter is composed of four layers with two layers of Nylon66 membrane (pore-size of 0.1 or 0.2 μm) sandwiched between two polyester microfiber layers. The 25-cm long filter cartridge was first cut into 2-cm thick discs using a bleached bandsaw and then further diced into ~1×2 cm^2^ slices with a flamed razor in a sterile laminar flow hood. Filter slices from one-eighth of each disc were stored in 15-mL polystyrene Falcon tubes according to membrane type. A short-clip of this procedure is available on YouTube (https://www.youtube.com/watch?v=_we9SOYJ660).

A protocol has been developed to isolate DNA, RNA and proteins from the exact same sample (the outer polyester microfiber layer and the two Nylon66 layers). Reaction tubes were kept on ice during the course of extraction, unless otherwise specified, in order to minimize degradation of molecules (especially RNA). Only the procedure of DNA extraction is described here. Microbial cells were lysed in 2× CTAB lysis buffer containing lysozyme (5 mg/mL final concentration) and Proteinase K (0.2 mg/mL final concentration) with a 30-min incubation at 60°C, followed by phenol/chloroform extraction. Phenol/chloroform/isoamyl alcohol (25:24:1) was added to the lysate (4:5 v/v). The mixture was placed into the 60°C-water bath for 1 min and then an ice-bath for 5 min before centrifugation at 4300× g for 10 min at room temperature. Nucleic acids (DNA and RNA) were precipitated by adding isopropanol (1:1 v/v), incubating on ice for 30 min and centrifuging at 4300× g for 15 min at room temperature. Supernatants were decanted, and the pellets were rinsed using pre-chilled 75% ethanol. The air-dried pellets were re-suspended in 1× TE-buffer (Tris-EDTA, pH = 8) and stored in 1.5 mL eppendorf tubes at −20°C until further processing.

An aliquot of the nucleic acids was treated with RNase A (10 μg/mL final concentration) for 30 min at 37°C. NaCl (0.1 M final concentration) and two-volumes of absolute ethanol were added. The mixture was incubated at −20°C for 30 min and centrifuged for 30 min at 11,500× g at room temperature to collect DNA.

### Sequencing

DNA samples (BE2011, BE2012, DR5, FI88, and TT109) were sequenced at National Center for Genome Resources, Santa Fe, NM. The KAPA High Throughput Library Preparation Kit (KAPA Biosystems) was used to prepare metagenome libraries with an insert size of ~280 bp using 500 ng of each DNA sample, and followed by 8 PCR cycles. Paired-end sequencing (2× 100 nt) was performed on an Illumina HiSeq 2000.

Metagenomic libraries with an insert size of ~170 bp were prepared using the Nugen Ultralow Ovation system (NuGen Technologies) for samples MM5 and TT107 (~2 ng of DNA per sample). Eighteen PCR cycles were applied to generate sufficient materials for sequencing. Paired-end sequencing (2× 100 nt) was performed on an Illumina HiSeq 1000 at Marine Biological Laboratory, Woods Hole, MA.

### Sequence assembly and annotation

For samples BE2011, BE2012, DR5, FI88, and TT109, k-mer spectra were created prior to assembly using Genome Assembly Evaluation Metrics and Reporting (GAEMR v1.0.1) to observe k–mer profiles. Samples were assembled using the ABySS v1.3.6 assembler on an MPI enabled cluster with k-mers ranging from 51 to 100 nt (Simpson et al., 2009). Contigs generated from different k-mers were pooled for completeness (Robertson et al., 2010) and processed in the Cap3 OLC assembler (Huang and Madan, 1999) with high overlap identity and reduced overhang stringency. Although Cap3 is traditionally an Expressed Sequence Tag (EST) assembler, it worked well for our samples where the input space was largely contiguous and the data sets were small (millions of bases). A database of all bacterial and archaeal proteins was retrieved from the NCBI ftp server (all.faa.tgz) and was clustered at 98% global identity using CD-HIT-EST v4.6.1 (Li and Godzik, 2006; Fu et al., 2012). Alignments were performed using NCBI-blast+ v2.2.28, and hereafter blast (Camacho et al., 2009). Open reading frames (ORFs) were predicted using the EMBOSS toolkit v6.4.0 with translation table 11 and peptides were called between start and stop codons in all frames (Rice et al., 2000). The predicted peptides were surveyed using the NCBI-all database and blastp. Hits were filtered based on 50% identity and at least 50% of the ORF covered. The predicted peptides were screened against Pfam-A v27.0 using HMMER3 using the default thresholds (Durbin et al., 1998; Eddy, 2008; Punta et al., 2012).

For sample TT107, paired-end reads were joined, quality-controlled (QC) and annotated using the standard MG-RAST metagenomic pipeline (http://metagenomics.anl.gov; Meyer et al., 2008). The post-QC reads were downloaded and assembled using IDBA-ud (Peng et al., 2011). ORFs in contigs longer than 200 nt were predicted using Prodigal (Hyatt et al., 2010) and then clustered at 90% identity using CD-HIT-EST. The representative ORFs (the longest in the cluster) were annotated against the m5nr database (ftp://ftp.metagenomics.anl.gov/data/M5nr/) using blastp algorithm to obtain the top ten closest hits (option:–max_target_seqs 10) with a maximum *e*-value threshold of 10^−5^. A consensus protein annotation was then selected using the majority rule.

Raw reads of sample MM5 were processed following the method of Howe et al. (2014) and the assembly protocol can be found at http://khmer-protocols.Readthedocs.org/en/v0.8.4/metagenomics/. In brief, low-quality reads were discarded. Post-QC reads were filtered by coverage (normalized) and assembled using various assemblers. Assembled contigs were uploaded to MG-RAST for annotation.

### Search for homologous ORFs

Since the seven metagenomes were annotated differently, a two-step approach was used to collect homologous ORFs that were shared. First, the pfam annotations of ORFs were screened for a list of key enzymes/functional genes in CH_4_, S, and N metabolisms (Supplementary Table 1) by custom scripting. ORFs of these enzymes/functional genes that were shared among samples BE2011, BE2012, DR5, FI88, and TT109 were collected because their contigs were annotated by the same pipeline. Then, the resultant common enzymes/functional genes were searched in samples TT107 and MM5 based on the MG-RAST annotation of ORFs. Datasets were created for each common functional gene, and those containing at least one sequence from each metagenome were subjected to curation and phylogenetic analyses.

Homologous ORFs in samples BE2011, BE2012, DR5, FI88, and TT109 were also identified based on the accession number of their best hit in the search against the NCBI non-redundant protein database (NR; downloaded on May 8, 2013), or hereafter called NR-best hits. Among the 81 accession numbers that were shared among samples BE2011, BE2012, DR5, FI88, and TT109, 9 belonged to *Thermodesulfovibrio yellowstonii*. However, only ORFs annotated as pyruvate oxidoreductase, alpha, beta and gamma subunits (*Por*A, *Por*B, and *Por*C, respectively), were detected in samples TT107 and MM5. The evolutionary relationship of these *T. yellowstonii*-like *Por* genes was further studied.

### Curation

For confident function assignments, all putative homologous ORFs with a minimum length of 50 amino acids (aa) were curated to remove false positives. Putative homologous ORFs were searched against NCBI NR using blastp algorithm to obtain the top 10 closest hits. The blastp result of some putative homologous ORFs was not consistent with the protein identity suggested by the analyses against the pfam database. The identity of all putative homologous ORFs was evaluated based on blastp results (alignment length, *e*-value and bitscore) and multiple-sequence alignments (MSA). For each functional gene, MSAs were generated for putative homologous ORFs from this study and the top 10 closest hits of each ORF. In most cases, reference sequences annotated as “putative” or “hypothetical” proteins were excluded. Jalview (Waterhouse et al., 2009) was used as the workbench for MSAs and manual editing to correct alignment errors. Alignment strategies MUSCLE (Edgar, 2004) and ClustalW (Thompson et al., 1994) were tried and ClustalW-MSAs were chosen. Alignments were carefully examined to remove poorly aligned sequences (both reference sequences and this study's ORFs). ClustalW-MSAs were then re-generated and edited for phylogenetic analysis.

For an ORF of minimum length of 50 aa to be included for analysis, it was at least represented by 2 sequences (paired-end reads being joined together) or more (reads being assembled into longer contigs). Together with the careful curation step to discard sequences based on alignments, such selection would lead to underestimation of the discovered sequence diversity of the common functional genes, but these sequences are of high quality.

### Taxonomic analyses

The finalized homologous ORFs of each common functional gene were taxonomically assigned in accordance with the lineage of its NR-best hit to create the microbial profile at the phylum and genus level.

### Phylogenetic analyses

The discordance between phylogenies of 16S rRNA and functional genes as a result of HGT has been widely reported in the literature (e.g., for the *dsr*AB gene see Klein et al., 2001), which prompts cautious inferences between physiological features (decoded from functional genes) and 16S-rDNA-defined identity. A previously published Witwatersrand subsurface pan-genome showed that the *Nif*H gene of the firmicute *Candidatus Desulforudis audaxviator* has an archaeal origin (Chivian et al., 2008). Also, the public databases are skewed toward cultivated strains with environmental functional gene sequences either being underrepresented or whose taxonomy may have not been verified. Thus, microbial profiling of functional genes based on the taxonomy of the host microorganisms will be prone to error. Therefore, this study took a phylogeny approach that is less sensitive to the taxonomic identity confusion caused by HGT and is more powerful than taxonomic profiling because the actual gene trees were studied.

For the MSA of each common functional gene, ambiguously aligned regions and positions with >50% of sequences containing a gap “-” were trimmed. Each trimmed MSA was then analyzed by ProtTest v3.0 (Darriba et al., 2011) to select the best evolutionary protein model based on the Bayesian Information Criterion (BIC). Maximum likelihood (ML) trees were constructed using the selected model using RAxML (Stamatakis, 2006), with 100 iterations for bootstrapping.

FastUnifrac (Hamady et al., 2009) was used to assess the phylogenetic relatedness of samples for each of the common functional genes based on topological distribution of the retrieved sequences on mid-point rooted ML trees. Clustering of sequences according to metagenome was evaluated by the parsimony test (*P*-test) (Martin, 2002) with the principle that fewer parsimonious changes are required to explain the clustering of sequences from a sample (on a tree) than a clade containing sequences from multiple samples. To circumvent the effect of uneven number of sequences representing the metagenomes on the observed clustering, the relationships between metagenomes were determined by Jackknife analysis that resampled randomly the minimum number of sequences among the samples (one for *Nar*V, *Nif*H, *Nif*D, *Nif*K, *Nif*E, and *Nif*N genes, three for PAPS reductase gene and four for NPD gene) for 1000 permutations.

Since *T. yellowstonii*-like *Por* genes originating from a single contig were identified in five samples, and ProtTest analyses of individual *Por* gene dataset selected the same evolutionary model (LG+I+G) for *Por*C, *Por*A, and *Por*B genes, phylogenetic analysis was performed on *Por*C-AB operon (the natural cluster is *Por*CDAB). In the cases of DR5 and MM5, the *T. yellowstonii*-like *Por* genes were detected in multiple contigs and they did not overlap. Since the *Por* gene segments of these two samples showed consistent relationship with other samples as suggested by their positions on the ML trees of *Por*A, *Por*B, and *Por*C genes, they were assorted accordingly to form the *Por*C-AB-like operons for samples DR5 and MM5. Reference sequences of *Por* genes were downloaded from the NCBI website and *Por*C-AB operons were constructed manually. Bayesian likelihood trees of *Por*C-AB operons were built using MrBayes (Ronquist et al., 2012) with mixed protein models. Two independent runs were performed and each with 1,000,000 generations. Topological convergence of all trees was assessed based on an average standard deviation of split frequencies, and which oscillated between 0.03 and 0.01. The consensus tree was constructed with the first 25% of trees discarded (the default burn-in value).

### Statistical analyses

All statistic analyses were performed in R (www.r-project.org). Principle component analysis (PCA) of environmental parameters was performed using “*prcomp*” in the “stat” package. Missing values of physical-chemical parameters were filled using the mean of the values available. Function “*vegdist*” in “vegan” package was used to calculate dissimilarity matrices for taxonomic diversity at the phylum and genus level using Bray-Curtis distance and for environmental parameters using Euclidean distance. Functions “*adonis*” and “*betadisper*” in “vegan” package were used to perform PERMANOVA and PERMDISP analyses on taxonomic diversity data using 999 permutations (Anderson and Walsh, 2013). Geographic distance matrix was calculated from longitude and latitude coordinates using the great circle method by the “fields” package. Metagenomes were compared in pairs and the unique branches of a metagenome were scored to generate unweighted pair-wise UniFrac distance matrices. Unweighted pair-wise UniFrac distance was used because it takes into account the evolutionary relationship (depicted on ML trees) of the sequences in the communities being compared and the statistical values have been shown to correlate with the actual distance between simulated communities, although the linearity has shown to be sensitive to sampling effort (Schloss, 2008). Mantel tests comparing biological distance matrices with environmental and geographic distance matrices were computed using Spearman's rank correlation and 999 permutations in the “vegan” package. Similarly, biological distance matrices were compared to the pair-wise Euclidean distances computed from depth and groundwater residence time of the samples.

### Sequence availability

All unassembled metagenomic data are accessible on MG-RAST under the MG-RAST ID numbers as follows: BE2011 (4536100.3), BE2012 (4536472.3), DR5 (4536473.3), FI88 (4536074.3), TT107 (4529964.3), TT109 (4536476.3), and MM5 (4529965.3). Curated amino acid sequences of the functional genes used in this study are available in the Supplementary Material.

## Results

### Fracture water geochemistry and residence time

**Figure 1:**
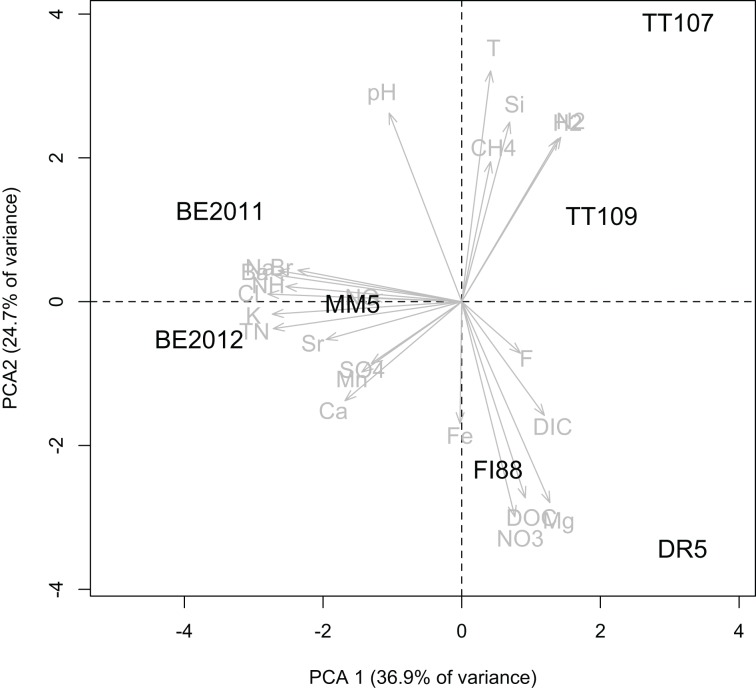
**Principle component analysis of physical and chemical characteristics of the fracture water samples**.

The fracture water samples had varying water chemistry (Table 1). PCA analysis showed that they were geochemically distinctive due to a combination of measured physical-chemical components (Figure 1). TT water samples were hotter and contained higher concentrations of dissolved CH_4_, H_2_, and N_2_. Samples from DR and FI were distinct based on the higher dissolved DOC, DIC, NO^−^_3_, and Mg^2+^ concentrations. The water samples from BE, MM and FI yielded δ^18^O and δ^2^H values that are similar to each other with an average value of −7 and −43‰, respectively, and all falling on the Global Meteroic Water Line (GMWL), as previously noted for BE and MM (Ward et al., 2004). The water samples from DR and TT yielded δ^18^O and δ^2^H values that are similar to each other with an average value of −5 and −24‰, respectively, which are only slightly elevated above the GMWL. The subsurface residence times are consistent with the isotopic signatures that indicate paleometeroic water, with the fracture water from DR and TT ranging from 1 to 24 kyr, whereas the residence times for the fracture water from BE, MM, and FI are potentially older, being at least 40 kyr.

### Shared functional genes

On average, 62 ± 23 × 10^6^ sequences of mean length of 106–108 bp were obtained per sample, with the least from BE2011 (46 × 10^6^ sequences) and the most from TT107 (113 × 10^6^ sequences). Based on the screening of samples BE2011, BE2011, DR5, FI88, and MM5, 13 functional genes were found in common: trimethylamine methyltransferases (*Mtt*B); dissimilatory sulfite reductase, delta subunit (*Dsr*D); phosphoadenylyl-sulfate (PAPS) reductase; respiratory (cryptic) nitrate reductase 2, gamma subunit and assembly co-factor (*Nar*V and *Nar*J, respectively); nitropropane dioxygenase (NPD); nitrogenase reductase (*Nif*H); nitrogenase Mo-Fe protein, alpha and beta subunit (*Nif*D and *Nif*K, respectively); nitrogenase FeMo cofactors (*Nif*E and *Nif*N); nitrite/sulphite reductase (*Nir*/*Sir*) family; and formate-nitrite transporter (FNT) family. The addition of samples TT107 and MM5 shortened the list to eight common functional genes (*Nar*V, NPD, PAPS reductase, *Nif*H, *Nif*D, *Nif*K, *Nif*E, and *Nif*N). Interestingly, seven of them are involved in N metabolism and five of these encode nitrogenase and associated proteins. Fewer functional genes related to S and CH_4_ metabolisms were shared among the studied samples.

### Taxonomic diversity

**Figure 2:**
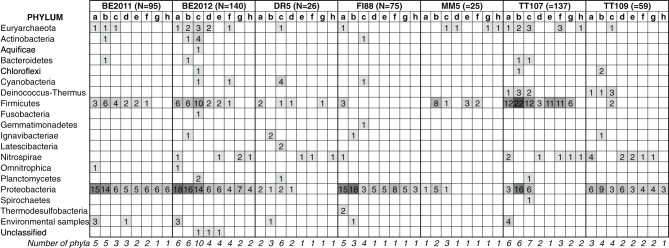
**Phylum-level taxonomic distribution of eight common functional genes detected in the subsurface metagenomes**. *N* denotes the total number of gene sequences detected in the metagenomes. The taxonomic classification of these sequences was assigned based on the lineage of their NR-best hit. The common functional genes are denoted by letters: a: *Nar*V gene; b: NPD gene; c: PAPS reductase gene; d: *Nif*H gene; e: *Nif*D gene; f: *Nif*K gene; g: *Nif*E gene; h: *Nif*N gene. Sequence counts were also overlain with scaled color intensity for visual effect. The last row gives the number of phyla represented by each common functional gene.

Taxonomic distribution of the eight common functional genes was summarized at the phylum and genus level. In total, one archaeal and 18 bacterial phyla spanning 126 genera were detected (Figure 2 and Supplementary Figure 1). The total number of gene sequences as well as the taxonomic richness of each of the common functional genes varied. PAPS reductases were encoded by a diverse microbial genome bank at the phylum level (Figure 2) whereas NPD had the greatest number of gene variants at the genus level (Supplementary Figure 1). Although Proteobacteria and Firmicutes were the dominant hosts of these common functional genes, pair-wise comparisons between metagenomes showed that the phylum-level diversity represented by these common functional genes differed from each other. *Nar*V, PAPS reductase and NPD related to the phylum Deinococcus-Thermus were unique to TT Au mines, the two hottest sites.

**Figure 3:**
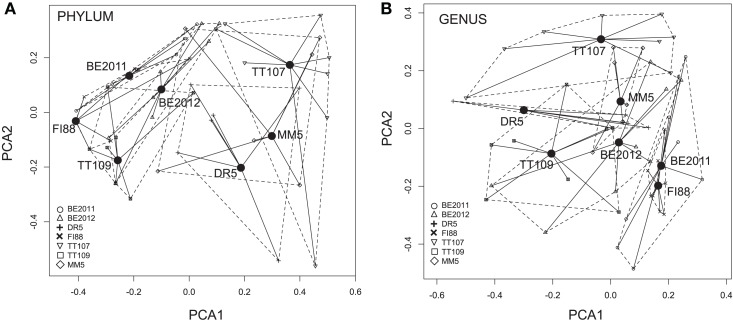
**Multi-dimensional scaling plots of dissimilarity in microbial compositions represented by the eight common functional genes within and between metagenomes at the phylum (A) and genus (B) level**. Filled circles denote the location of the centroid of each metagenomes. The closer the centroids, the more similar are the taxonomic compositions of the represented metagenomes. Each centroid is joined to the eight common functional genes (denoted by metagenome-specific symbols) by black lines that represent the distance between the centroid and each gene. The dashed lines encapsulate the “distance space” of each metagenome. The size and shape of the “distance space” indicate within-sample variance in the taxonomic profiles. The inter-sample variances were significantly different at the phylum level but not at the genus level.

At the phylum level, PERMDISP analysis indicated that the inter-sample variance in the taxonomic diversity represented by the common functional genes was significantly different among these metagenomes (*F* = 4.23, *p* = 0.002) (Figure 3A). Microbial profiles of all 5 *Nif* genes in metagenome FI88 belonged to the phylum Proteobacteria whereas, most of the common functional genes detected in the metagenomes BE and TT represented 2–10 phyla (Figure 2). PERMANOVA analysis indicated that the metagenomes were taxonomically different (*F* = 5.25, *p* = 0.001). This result, however, needs to be taken with caution because of the unequal variance between metagenomes. Some of the common functional genes belonging to the same phylum (and even genus) occurred in multiple samples (Figure 2). For examples, a suite of common functional genes from Proteobacteria (*Azoarcus*, *Candidatus Accumulibacter*, and *Dechloromonas*), Firmicutes (*Ca. Desulforudis* and *Thermincola*), and Nitrospirae (*Thermodesulfovibrio*) were present in at least two metagenomes, with a nearly complete set from *Ca. Desulforudis* being detected in three metagenomes (BE2011, BE2012, and TT107).

The taxonomic composition of common functional genes also differed among metagenomes at the genus-level (PERMANOVA, *F* = 2.93, *p* = 0.001), and with inter-sample variances not being statistically different (PERMDISP, *F* = 0.58, *p* = 0.751) (Figure 3B). The more equivalent variances between metagenomes could be explained by the progressively fewer groups being shared by metagenomes at higher taxonomic resolution as one goes from phylum to genus. Evidence of prevalence of different microbial genera in different samples was found (Supplementary Figure 1). For instance, genes from firmicutes *Desulfotomaculum* and *Desulfurispora* were detected mainly in sample TT107. Genes related to multiple methanogenic genera were more concentrated in sample BE2012 but none was detected in sample FI88. In addition, most *Nif* gene variants belonging to *Methanobacterium* and *Methanothermobacter* were present in samples MM5 and TT107, respectively. Moreover, *Nif*H, *Nif*D, and *Nif*K genes were represented in 20 contigs, which further illustrates that the nitrogenases in our samples were hosted by different members of Proteobacteria and Firmicutes (Supplementary Table 2).

Mantel test results indicated that the taxonomic dissimilarity between samples for each common functional gene, at either phylum or genus level, do not have statistically significant correlations with longitude and latitude coordinates, physical and chemical data, depth and groundwater residence time of each site (Table 2).

**Table 2:** **Spearman correlation coefficients and significance values of Mantel tests between biological distance matrices and distance matrices of abiotic factors**.

	**Mantel test against geographical distance**	**Mantel test against environmental distance**	**Mantel test against depth gradient**	**Mantel test against age difference**
**TAXONOMIC DIVERSITY**				
Phylum-level	*r* = −0.2605 (*p* = 0.904)	*r* = −0.3247 (*p* = 0.919)	*r* = 0.04163 (*p* = 0.355)	*r* = −0.1187 (*p* = 0.608)
Genus-level	*r* = −0.2142 (*p* = 0.791)	*r* = −0.1267 (*p* = 0.681)	*r* = 0.1171 (*p* = 0.287)	*r* = 0.3679 (*p* = 0.161)
**PHYLOGENETIC DIVERSITY**				
*Nar*V gene	*r* = 0.1515 (*p* = 0.238)	*r* = −0.2377 (*p* = 0.862)	*r* = 0.01691 (*p* = 0.39)	*r* = 0.2595 (*p* = 0.209)
NPD gene	*r* = 0.001306 (*p* = 0.51)	*r* = 0.2208 (*p* = 0.193)	*r* = −0.5528 (*p* = 0.592)	*r* = 0.05543 (*p* = 0.326)
PAPS gene	*r* = 0.02677 (*p* = 0.447)	*r* = 0.01688 (*p* = 0.499)	*r* = 0.2276 (*p* = 0.171)	*r* = −0.1069 (*p* = 0.664)
*Nif*D gene	*r* = −0.1188 (*p* = 0.686)	*r* = 0.02857 (*p* = 0.387)	*r* = −0.06829 (*p* = 0.529)	*r* = 0.08803 (*p* = 0.32)
*Nif*E gene	*r* = 0.1196 (*p* = 0.333)	*r* = −0.3317 (*p* = 0.903)	*r* = 0.075 (*p* = 0.339)	*r* = 0.2019 (*p* = 0.234)
*Nif*H gene	*r* = 0.5359 (*p* = 0.023)^*^	*r* = 0.1837 (*p* = 0.232)	*r* = −0.07504 (*p* = 0.568)	*r* = 0.2126 (*p* = 0.189)
*Nif*K gene	*r* = 0.3265 (*p* = 0.424)	*r* = −0.1649) (*p* = 0.745)	*r* = 0.132 (*p* = 0.256)	*r* = 0.2224 (*p* = 0.226)
*Nif*N gene	*r* = 0.1485 (*p* = 0.275)	*r* = −3438 (*p* = 0.938)	*r* = −0.0563 (*p* = 0.524)	*r* = −0.01838 (*p* = 0.396)

Values were derived from 999 permutations.

* Indicates tests that were statistically significant at the alpha level of 0.05.

### Phylogenetic relatedness

**Figure 4:**
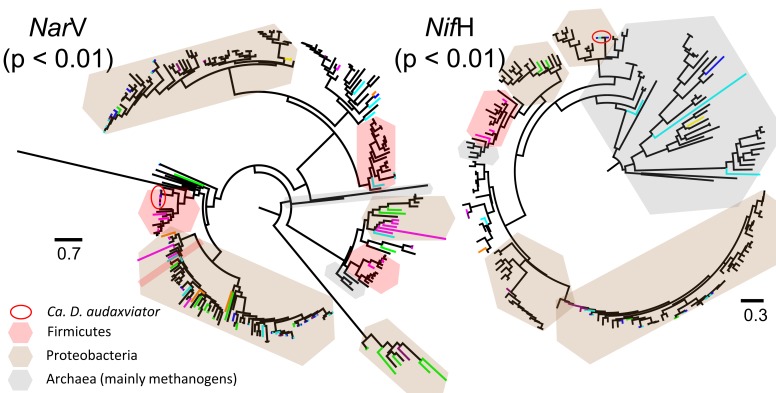
**Maximum likelihood trees of deduced amino acid sequences of *Nar*V and *Nif*H genes detected in assembled metagenomes**. Sequences from this study are highlighted by color lines, with blue for BE2011, cyan for BE2012, orange for DR5, green for FI88, magenta for TT107, dark purple for TT109, and yellow for MM5. *P*-test significance values of unweighted UniFrac distances among metagenomes are given. Scale bars represent the amino acid substitution rate per site. Number of taxa and characters, and amino acid evolutionary models used are as follow: *Nar*V gene: 273, 188, LG+G; NPD gene: 584, 248, LG+G; PAPS reductase gene: 479, 159, LG+G; *Nif*H gene: 191, 268, LG+I+G; *Nif*D gene: 154, 415, LG+I+G; *Nif*K gene: 196, 419, LG+I+G; *Nif*E gene: 137, 427, LG+I+G; and *Nif*N gene: 84, 423, LG+I+G.

Overall, our sequences encoding each common functional gene were phylogenetically diverse, as they spread across the respective gene trees (examples are given in Figure 4). Noteworthy is that the gene variants from the studied metagenomes inter-dispersed on the gene tree with formation of sample-specific clusters. The close relationship of sequences from the same metagenome was supported by *P*-tests (*p* < 0.01 for all common functional genes). In the case of *Nif*H gene, the *Ca. D. audaxviator*-like *Nif*H genes detected in our samples were closely affiliated to sequences belonging to the archaeal order Methanobacteriales (Figure 4), as previously reported (Chivian et al., 2008). This example illustrated the value of phylogeny-based methods in assessing microbial diversity.

**Figure 5:**
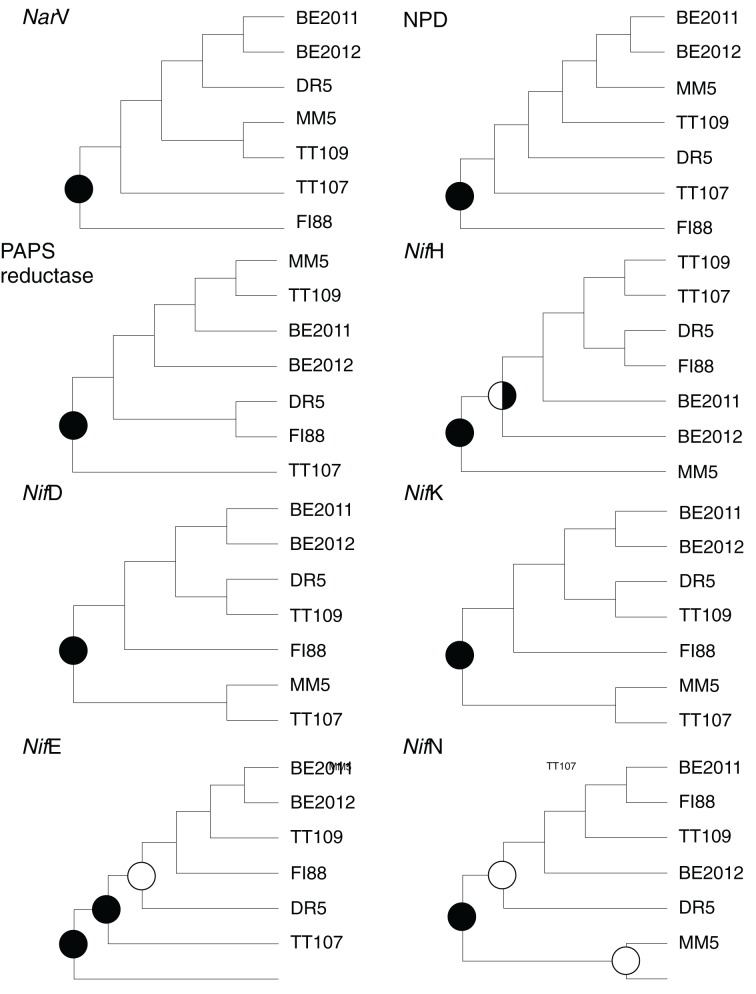
**Phylogenetic relatedness of the studied metagnomes revealed by each of the common functional genes**. Branch support of the dendrograms was derived from 1000 permutations of Jackknife resampling. Nodes supported by 50–69% (open circles), 70–89% (half-filled circles), and 90–100% (filled circles) of the permutated trees are indicated.

The robustness of branching in the eight common functional genes for the studied metagenomes (Figure 5) was the strongest at the root that set apart the *Nar*V and NPD gene communities of sample FI88 from the others, the PAPS reductase gene community of sample TT107 from the others, the *Nif*H gene communities of sample MM5 from the others, and the remaining *Nif* gene communities of samples MM5 and TT107 from the others. However, the weaker branching support at the internal nodes of the dendrograms indicated that the trees obtained through the 1000 Jackknife resampling were not reproducible. It was therefore impossible to resolve the precise phylogenetic relationships among the seven metagenomes based upon these eight common functional genes.

The unweighted pair-wise UniFrac distance matrices calculated from the real gene trees of the common functional genes were compared to environmental and geographical distance matrices. Similar to the results obtained from taxonomic diversity, the genealogical distance of the common functional genes did not correlate well with the distances computed from any of the environmental factors, geographic distance, depth and groundwater residence time, except for one case (*Nif*H gene ~geographical distance) (Table 2).

**Figure 6:**
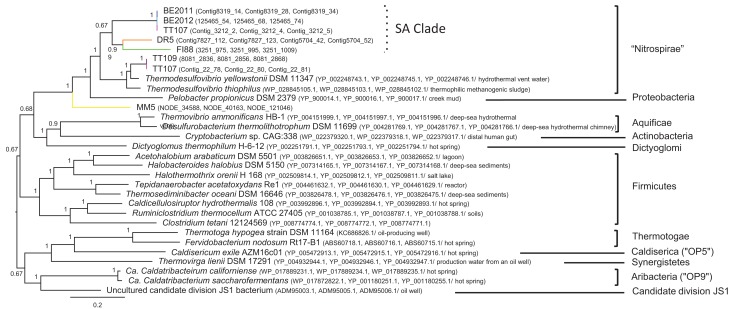
**Bayesian likelihood tree of deduced amino acid sequences of the *Por*C-AB operon**. The same color scheme as in Figure 4 is used to highlight our sequences. Our metagenomes are followed by contig IDs, whereas reference taxa are suffixed by GenBank accession numbers and, if available, the source of isolates. Phyla are given to the right of the tree. Nodes are supported by posterior probability and scale bar represents the amino acid substitution rate per site.

The *Thermodesulfovibrio*-like *Por*C-AB gene sequences recovered from the metagenomes did not form a sub-clade collectively within *Thermodesulfovibrio* spp. (Figure 6). The phylotype from sample BE2011 clustered more closely with that from samples BE2012 and TT107, than with that from samples DR5 and FI88. They formed a clade, which will be referred as South African Clade, or SA Clade. The SA Clade formed a well-supported clade separated from that containing the phylotypes detected in samples TT107 and TT109, *T. yellowstonii* and *T. thiophilus*. It is uncertain whether the SA Clade warrants being a novel taxonomic group at this moment, and therefore, it is assigned to the best-associated phylum, Nitrospirae. This “Nitrospirae” clade shared with the delta-proteobacterium *Pelobacter propionicus* the common ancestor that had diverged from the phylotype detected in sample MM5.

## Discussion

### N-cycling pathways selected by terrestrial subsurface environments

Among the genes in CH_4_, S, and N metabolisms being screened, the dominance of N-cycling genes in the common functional gene pool of the metagenomes implies that some N-metabolizing pathways or microorganisms with the potential to perform such processes may be preferentially selected by deep subsurface habitats. CH_4_ production and oxidation, and S reduction in subsurface habitats have been investigated using isotope geochemistry (e.g., Ward et al., 2004; Kieft et al., 2005; Onstott et al., 2006) and molecular microbiology (e.g., Moser et al., 2005; Pfiffner et al., 2006; Davidson et al., 2011; Itävaara et al., 2011; Lever et al., 2013; Purkamo et al., 2013). Comparatively, subsurface N cycling has received little attention. Transcripts coding for respiratory nitrate reductases (*Nar*) were expressed by alpha- and beta-Proteobacteria in deep-sea sediments where nitrate concentrations were below detection (Orsi et al., 2013). Denitrification at deep terrestrial subsurface sites in South Africa, including the Witwatersrand Basin, was suggested by enriched δ^18^O-NO^−^_3_ and δ^15^N-NO^−^_3_ values in fracture waters (Silver et al., 2012). N_2_ fixation was inferred to occur for *Ca. D. audaxviator*, based upon the presence of two types of nitrogenase encoded in its genome (Chivian et al., 2008).

Respiratory nitrate reduction is the first step of denitrification. *Nar*V encodes for the heme-Fe subunit (responsible for electron transport) of the cryptic isoenzyme (NRZ) of the membrane-bound nitrate reductase. Compared to the primary isoenzyme (NRA), NRZ accounts for only 10% of the total activity in *Escherichia coli* during exponential growth and is not induced by nitrate or anaerobic conditions (Moreno-Vivián et al., 1999). Instead, the regulatory mechanism includes the vegetative sigma factor *Rpo*S that controls gene expressions as *E. coli* cells transition into stationary phase or nutrient-poor conditions (Chang et al., 1999). It was shown that *Salmonella* wild-type cells grown under C starvation were able to tolerate thermal and low-acidity stresses better than the *Nar*Z-knock-out mutant cells (Spector et al., 1999). The detection of *Nar*V in our assembled metagenomes suggests that NRZ may have an important ecological function in our subsurface habitats where microbial growth is challenged, with protein turnover time on the order of 10^1−2^ years (Onstott et al., 2014). Nonetheless, respiratory nitrate reduction by NRA is not excluded because genes annotated as NRA components were also detected in the unassembled sequences (data not shown).

In addition to multiple N_2_-fixing genes detected in the metagenomes, genes encoding the enzyme subunits responsible for electron transfer (*Nif*H) and reduction activity (*Nif*D and *Nif*K) were assembled into single contigs (Supplementary Table 2). It is therefore concluded that microorganisms closely related to those listed in Supplementary Table 2 have the potential to express functional nitrogenases. Nitrogenase is better known in its role in N_2_ fixation (or N_2_ reduction to NH_3_ with H_2_ evolved as a byproduct), which is an energetically costly process that requires 16 ATP molecules per mole of N_2_ fixed (Postgate, 1982). But it also catalyzes reduction of other substrates. It catalyzes the reduction of H_2_O to H_2_ using H_2_ as a substrate, which means that high pH_2_ inhibits N_2_ fixation by nitrogenase (Guth and Burris, 1983). Vaughn and Burgess (1989) have shown that nitrogenases reduce one mole of NO^−^_2_ to NH_3_ *in vitro* in the presence of S_2_O^2−^_4_ and Mg-ATP molecules at the expense of 6 electrons. The possibility of nitrogenase acting as an assimilatory nitrite reductase in subsurface habitats cannot be completely ruled out because it is thermodynamically favorable, with ΔG varying from −320 to −370 kJ mol^−1^ of NO^−^_2_ (based on the equation in Guerro et al. (1981) and our geochemical data). Whether the nitrogenases in these subsurface systems fix N_2_ or reduce NO^−^_2_, or perhaps both, merits further investigation. Regardless, *Nir* genes encoding known nitrite reductases were detected in the unassembled sequences (data not shown), indicating the potential of nitrite reduction.

**Figure 7:**
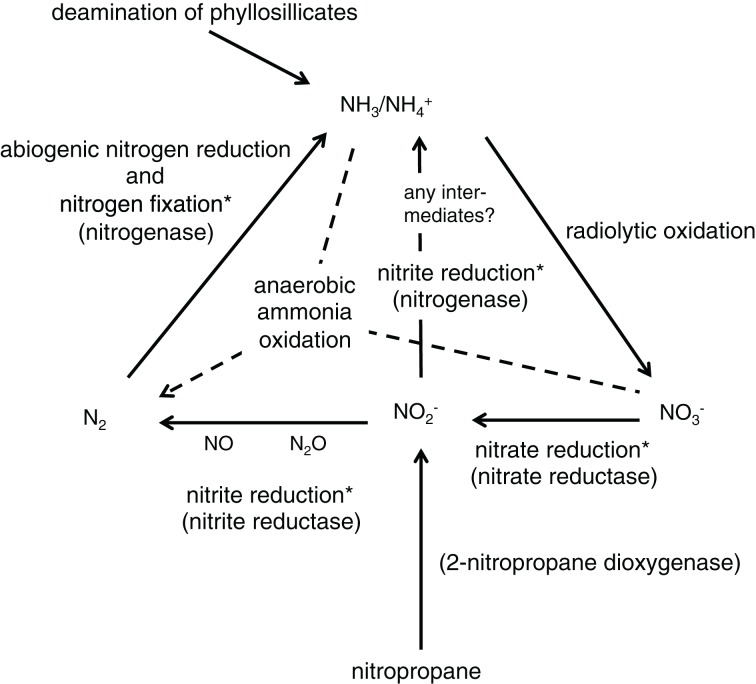
**Schematic diagram of the proposed N cycle in deep terrestrial subsurface sites in South Africa**. Solid lines indicate processes suggested by molecular (this study) and geochemistry analyses (Silver et al., 2012). Dashed lines indicate processes that are known in the global N cycle but their occurrence in deep terrestrial subsurface sites is yet to be proven. Asterisks denote microbially mediated processes.

Pairing the molecular data in this study with the geochemistry data in Silver et al. (2012) presents a closed N cycle in the deep terrestrial subsurface habitats (Figure 7). The majority of NH_3_/NH^+^_4_ resides in phyllosilicates and a minority likely comes from abiogenic and biogenic reduction of N_2_. NH_3_/NH^+^_4_ is transformed into NO^−^_3_ via radiolysis. NO^−^_3_ is then reduced by nitrate reductase (*Nar*) to NO^−^_2_. NO^−^_2_ is also formed from the reaction mediated by 2-nitropropane dioxygenase (NPD). The cycle is closed when NO^−^_2_ is reduced to NH_3_/NH^+^_4_ by nitrogenase (*Nif*) with unknown intermediates, or when NO^−^_2_ is first reduced by denitrification (*Nir*) to N_2_ that is further reduced by N_2_ fixation (*Nif*). The formation of N_2_ through anaerobic NH_3_ oxidation (anammox) is known to occur in marine and deep-sea sediments (Thamdrup and Dalsgaard, 2002; Glud et al., 2009), however, it has not yet been detected in the metagenomes reported here. The absence of hydrazine oxidoreductase (*hzs* genes) in these metagenomes agrees with the low abundance of Planctomycetes (1–3% of the bacterial communities) at the studied boreholes, as shown in a 16S rRNA gene amplicon study (Magnabosco et al., under review). The low number of *hzs* genes deposited in the NCBI database (230 protein sequences as of Aug 24, 2014), however, is also very likely to have reduced the ability to identify *hzs* genes in the metagenomic data.

### Diverse functional guilds

A functional guild is defined as a group of microorganisms that exploit the same substrate with the aid of the same enzyme. Our results showed that a taxonomically and phylogenetically diverse group of subsurface microorganisms has the metabolic potential to express the following common putative enzymes, namely nitrate reductases, 2-nitropane dioxygenases, PAPS reductases and nitrogenases (Figures 2, 4). Such biodiversity of functional guilds composed of multiple phyla appears to be greater than that reported from other extreme environments. A metagenome study of seven hot springs in Yellowstone National Park, USA documented key metabolic genes from up to five different archaeal orders (Inskeep et al., 2013). Putative hydrogenase genes belonging to less than five bacterial orders were detected in metagenomes from venting fluids at serpentinite sites at The Lost City and in spring water in Winter House Canyon (Brazelton et al., 2012). The caveat of making this comparison is that the overall community compositions in these sites are fundamentally different from the sites in this study.

There are three possible ways to explain the diverse functional guilds present at the sites of this study: (i) substrates for the common putative enzymes in deep terrestrial subsurface habitats may be readily available within the optimal range to support a diverse microbial group; (ii) competition for these substrates exists but has not been the main evolutionary force to drive elimination of species; or (iii) these enzymes (presumably the complete metabolic pathways represented by these enzymes) confer sufficient selective advantage that a variety of subsurface microorganisms retain or acquire the metabolic features to enhance self-sustainability and survivorship. For a community that relies on a keystone species for a specific metabolic role, extinction of that species would cause a breakdown of the food chain or metabolic network. Therefore, a diverse microbial community capable of performing the same function, such as those presented in this study, would greatly enhance the sustainability of the whole biome.

The occasions where the common functional traits in metagenomes DR5 (*Nif*D, *Nif*K, *Nif*E, and *Nif*N) and MM5 (*Nar*V, *Nif*H, *Nif*E, and *Nif*N) were only represented by a single sequence merits further evaluation. The total number of sequences retrieved from these two samples, 58.4 × 10^6^ and 58.9 × 10^6^ sequences, respectively, were not the fewest and were comparable to the average number of sequences of all metagenomes; thus insufficient sequencing effort should not be the sole explanation. These two metagenomes were handled, sequenced and assembled by completely different protocols and their initial annotation processes were also different. It does not appear that any of the technical approaches was particularly disadvantageous. The overall taxonomic diversity represented by the common functional traits of samples DR5 and MM5 were lower than that in the other samples, which suggests that the functional guilds of the common functional traits at these two sites are fundamentally less diverse. The criterion of selecting assembled contigs longer than 50 aa has put more weight on abundant taxa; as a result, microorganisms capable of performing the common functional traits may be less abundant in samples DR5 and MM5 than other samples. Since the single sequences of these two samples did not come from the same genus or phylum, it is not likely that only a single species possesses these functional traits. Rather, a combination of the aforementioned technical and intrinsic aspects may have exerted a compound effect, causing the lean sequence representation in samples DR5 and MM5. This reduced sequence representation may have diminished the ability to resolve the relative phylogenetic relatedness of the metagenomes in that it restricted the subsampling size in performing Jackknife analysis. Yet the reduced diversity and abundance of microorganisms capable of reducing nitrate (using *Nar* genes) and fixing N_2_ (using *Nif* genes) implies that, at sites DR5 and MM5, either the selective pressure for these functions was great enough to cause species elimination, or these functions were obsolete and nitrate reduction and N_2_ fixation were achieved by mechanisms other than the pathways dictated by *Nar* and *Nif* genes, respectively.

### Biogeography of common functional traits

The study sites are kilometers below land surface and kilometers to hundreds of kilometers apart, and each fracture water has distinct physical and chemical attributes. The similarity in the δ^18^O and δ^2^H values between water samples from DR5, TT107 and TT109 is consistent with their proximal geographic positions. The DR5 water sample was collected from the Transvaal dolomitic aquifer, which at this location is overlain by impermeable banded iron formation that serves as a confining layer. The dolomite aquifer in this region north of the Vaal River is subdivided into compartments by north-south striking dykes, and water sample from DR5 occurs within the Oberholzer compartment. Recharge for the Oberholzer compartment occurs in the dolomitic outcrops 20–30 km north followed by groundwater migration to the south where it encounters the cone of depression surrounding the DR mine (DWAF, 2006). The TT water samples were collected ~200 m apart within the Witwatersrand quartzite along the margins of Jeans Dyke, which is possibly Karoo in age. The different chemistry of the TT water samples from that of the dolomitic water, and the younger subsurface residence time for TT107, indicates that the fracture water at TT has not recharged downwards from the overlying dolomite aquifer. Instead, this water likely originates by recharge in non-dolomitic exposures >30 km to the north flowing along the margins of Jeans Dyke, which cuts across all Precambrian stratigraphy and structures toward the cone of depression surrounding the TT Au mines in this area.

The δ^18^O and δ^2^H values for BE326 and MM5 water samples are similar to those previously reported from the Welkom mining region, and although also on the GMWL, they are distinct from those lying on the meteoric water line from the northern and eastern margins of the Witwatersrand Basin (Ward et al., 2004; Onstott et al., 2006). Only two regions exist in South Africa with predicted δ^2^H values for precipitation that coincide with the very light −40 to −47‰ range observed for the Welkom fracture water samples, the Kalahari Desert and the Lesotho highlands (West et al., 2014). Given that the Welkom mining district lies south of the Vaal River at an elevation of 1370 m, it is more likely that groundwater recharge for this mining district occurs 150 km to the southeast in the mountains of Lesotho (at an elevation of ~2500 m), than from the Kalahari Desert (across the Vaal River to the north and at a lower elevation), or from the same recharge zone as DR and TT Au mines (on the northern Witwatersrand Basin, across the Vaal River and 250 km northeast at an elevation of only 1500 m). The long flow path from the Lesotho highlands could explain the older ages for the Welkom fracture water.

FI diamond mine lies north of the Vaal River at the top of the Ghaap Plateau. The dolomitic aquifer sits beneath banded iron formation. The recharge is considered to be local, although limited, through fractures in the iron formation and dolomite (DWAF, 2006). If this is true, then the mean ^81^Kr groundwater residence time of ~410 kyr age is suggestive of a very slow ground water migration or a mixture of younger and older water. The latter explanation is more likely since the geochemical composition of FI88 fracture water lies between that of the dolomitic Dr5 and the older, more saline BE and MM5 fracture water (Figure 1).

The isotopic and groundwater chemistry data suggest a spatial biogeography that DR5, TT107 and TT109 microbial communities should be most similar to one another, those of BE326 and MM5 should be most similar to each other and FI should be distinct from these two clusters. From a temporal perspective, samples DR5 and TT should be most representative of the recharging microbial communities, although the high temperatures of TT may result in greater communities divergence as a result of adaptive changes. On the other hand, the older residence times of water sample FI88, MM5, and BE326 suggest that microorganisms in these samples have had much longer time to evolve in response to the subsurface environment than have DR5 and TT samples.

Results of this study showed that the taxonomic and phylogenetic distributions of the common functional traits are distinctive for each sample and not correlated to the distance of separation between sites, the similarity in environmental characteristics, or the differences in groundwater residence time or depth. Only one exception exists with respect to this conclusion and that is the dissimilarity in *Nif*H communities being correlated positively with geographical distance (Mantel test, *r* = 0.5359). It is therefore interpreted that the assemblages of common functional traits in these terrestrial subsurface habitats displayed a high heterogeneity, yet the underlying drivers have not been identified.

Metabolic genes are non-neutral, i.e., they undergo greater selection. As these functional genes are common to all study sites, as previously discussed, they may already be the product of selection by factors other than the two most ecologically influential factors (physical distance and environmental features) usually reported in other environments (citations in the Introduction). Subterranean dispersal is very restricted and probably infrequent and slow compared to dispersion in surface habitats, and thus some communities may have been isolated for thousands of years. The biogeography of these common functional genes may thus have been subjected to selection by environmental features that were not measured, geological history, and biological connectivity (rather than being influenced solely by physical distance). In addition, HGT certainly complicates the biogeography of functional genes, particularly if gene transfer through HGT is more mobile than the transport of microbial cells. Recently, phage-transduction was shown to be frequent among diverse microbial recipients in river samples (Kenzaka et al., 2010). Through incubation experiments, the study also showed the gene transfer frequency remained at 10^−2−3^ per colony-forming unit at a range of the recipient cell concentration (10^3−8^ cells mL^−1^). Signs of phage-infection have been detected in samples from deep-sea sediments (Orcutt et al., 2011) and continental fracture fluids (Chivian et al., 2008; Nyyssönen et al., 2014). If the occurrence of phage-mediated HGT in the deep biosphere is frequent relative to any changes in the environmental state or microbial transport, then it might exert an unappreciated effect on biogeography by introducing genetic diversification. Microbial dispersal, selection pressure and the mechanism of genetic transfer certainly merit consideration in subsurface biogeography in general, as well as for phylogenetic studies of single gene or multi-genes, functional or non-functional genes.

### Subsurface relatives of thermophile T. yellowstonii

The *Por*C-AB genes detected in these samples were most similar to those of thermophilic sulfate-reducing bacteria, *Thermodesulfovibrio* spp. *T. yellowstonii* strain YP87 can grow on pyruvate as the electron donor and sulfate as the electron acceptor (Henry et al., 1994). This may involve the activity of pyruvate oxidoreductase (other synonyms: pyruvate synthase, pyruvate:ferredoxin oxidoreductase, pyruvate synthetase, pyruvic-ferredoxin oxidoreductase) that is known to catalyze catabolic and anabolic reactions of pyruvate (Furdui and Ragsdale, 2000). Some of the studied boreholes have a temperature within the growth temperature range of the type strain YP87 (40–70°C) (Henry et al., 1994), whereas the temperatures at where samples DR5 and FI88 (27–29°C) were collected are below the growth temperature range. However, strain YP87 has been shown to remain viable at 27°C for at least 1 year (Henry et al., 1994). The clustering of subsurface *Por*C-AB phylotypes suggests a dependence on site temperature, although the second TT107 phylotype was more affiliated with the BE phylotypes (Figure 6). It is possible that the SA Clade may represent mesophilic members or relatives of *Thermodesulfovibrio*. It is also possible that this study's *Thermodesulfovibrio*-like phylotypes would exist at different states of activity at the respective borehole as a result of the temperature difference.

The closest phylogenetic affiliate of the phylum Nitrospirae is delta-Proteobacteria as suggested by the phylogeny of 16S rRNA genes (Teske et al., 1994; Castro et al., 2000) and by genome organization and gene arrangements (Kunisawa, 2010). In agreement with these observations, analysis of *Por*C-AB genes also indicates that Nitrospirae and Proteobacteria share an evolutionary relationship exclusive of other phyla. The divergence of the MM5 phylotype from the common ancestor of *Thermodesulfovibrio* spp. and *P. propionicus* was highly robust, which strongly points to an ancestral state of its genetic content. A more in-depth investigation of the phylogenetic relationship of these subsurface phylotypes, in particular the one from sample MM5, with those of Nitrospirae and Proteobacteria would shed light on the evolution of these phyla. This result also suggests that subsurface habitats have preserved microorganisms that provide valuable genetic information on the origin and evolution of prokaryotes.

## Conclusion

These findings have proposed how N may be cycled within the South African continental crust. Diverse functional guilds were detected in subsurface metagenomes, however, the heterogeneity in taxonomically- or phylogenetically-defined diversity does not correlate with geographical distance, environmental parameters and the subsurface residence time of the fracture water. Since this study focused on a select subset of functional genes, the biogeographic distribution of total functional genes may show a different relationship with the shaping forces, which merits further study in the future. The exercise of searching for common functional genes facilitated an initial attempt to explore metagenomic data for the investigation of the evolutionary relationship between surface and subsurface genes and microorganisms.

### Conflict of interest statement

The Review Editor, Karen Lloyd, declares that, despite having collaborated with author, Tullis C. Onstott, the review process was handled objectively. The authors declare that the research was conducted in the absence of any commercial or financial relationships that could be construed as a potential conflict of interest.
