# Supplementary material for: Phylogeny and phylogeography of functional genes shared among seven terrestrial subsurface metagenomes reveal N-cycling and microbial evolutionary relationships
Source: Front Microbiol. 2014 Oct 31;5:531. doi: 10.3389/fmicb.2014.00531 (PMC4215791; doi:10.3389/fmicb.2014.00531)
Supplement: Supplementary file 1 [file Table1.DOCX]

**Supplementary materials**

This document contains:

Supplementary Table 1. List of key enzymes and functional genes queried in the assembled metagenomes by string search.

Supplementary Table 2. Contigs that contain *Nif*H, *Nif*D and *Nif*K gene sequences

Supplementary Figure 1. Genus-level taxonomic distribution of 8 common functional genes detected in the subsurface metagenomes. N denotes the total number of gene sequences detected in the metagenomes. The taxonomic classification of these sequences was assigned based on the lineage of their NR-best hit. The common functional genes are denoted by letters: a: NarV gene; b: NPD gene; c: PAPS reductase gene; d: NifH gene; e: NifD gene; f: NifK gene; g: NifE gene; h: NifN gene. Sequence counts were also overlain with scaled color intensity for visual effect (blue for low values and red for high values). The last row gives the number of phyla represented by each common functional gene. This figure is best viewed with at least 200% magnification.

Curated amino acid sequence of ORFs analyzed in this study are provided in a separate file:

Filename – Lau et al _supplementary_seqs.txt

File size: 204 KB

Supplementary Table 1. List of key enzymes and functional genes queried in the assembled metagenomes by string search.

|  | Enzyme | Gene |
| --- | --- | --- |
| CH_4_ metabolism | Methane monooxygenase | mmoX, mmoY, mmoZ, mmoB, mmoC, mmoD, pmoA, pmoB, pmoC |
|  | Coenzyme M methyltransferase | mtaA, mtaB, mtaC, mtbA, mtbB, mtbC |
|  | Methanol dehydrogenase | Mdh1, mxaF, mxaJ, mxaG, mdh2, mxal, mxaA, mxaC, mxaK, mxaL, mxaD, |
|  | Methyl-coenzyme M reductase | mcrA, mcrB, mcrC, mcrD, mcrG |
|  | Heterodisulfide reductase | hdrA, hdrB, hdrC, hdrD, hdrE |
|  | Tetrahydromethanopterin S-methyltransferase | mtrA, mtrA2, mtrB, mtrB2, mtrC, mtrC, mtrE, mtrF, mtrG, mtrH |
|  | Trimethylamine methyltransferase | mttB |
|  | Trimethylamine corrinoid | mttC |
|  | Methylamine methyltransferase | mtmB |
| S metabolism | Sulfur transportation | cysA, cysP, sbp, cysU, cysW |
|  | Sulfur oxidation | soxA, soxB, soxX, soxY, soxZ |
|  | Sulfane dehydrogenase | soxC |
|  | Thiosulfate/3-mercaptopyruvate sulfurtransferase | TST, MPSt, sseA |
|  | Thiosulfate sulfurtransferase | glpE |
|  | Thiosulfate reductase | phsB, phsC |
|  | Polysulfide reductase | phsA, psrA |
|  | Sulfide-quinone oxidoreductase (Sulfide:quinone oxidoreductase) | sqr |
|  | Sulfur reductase | sreA, sreB, sreC |
|  | Sulfur oxygenase | Sor |
|  | Sulfite reductase | cysJ, cysI, sir, dsrA, dsrB |
|  | Anaerobic sulfite reductase | aasrA, asrB, asrC |
|  | Alkanesulfonate monooxygenase | ssuD |
|  | Sulfite dehydrogenase |  |
|  | Sulfide oxidase | suoX |
|  | Methylthio-coenzyme M methyltransferase (methylthio:coenzyme M methyltransferase) | mtsA, mtsB |
|  | Dimenthyl-sulfide monooxygenase | dmoA |
|  | Dimenthylpropiothetin dethiomethylase | dddL |
|  | Anaerobic dimethyl sulfoxide reductase | dmsA, dmsB, dmsC |
|  | Dimethylsulfoniopropionate demethylase | dmdA |
|  | Dimethylsulfone reductase (dimethyl sulfone reductase) |  |
|  | Dimethylsulfone monooxygenase (dimethyl sulfone monooxygenase) | sfnG |
|  | Methanesulfonate monooxygenase | msmA, msmB |
|  | Phosphadenosine phosphosulfate reductase | cysH |
|  | Adenylylsulfate reductase (adynylyl-sulfate reductase) | aprA, aprB, APR |
|  | Adenosine 5’-phosphosulfate reductase |  |
|  | Adenylysulfatase |  |
|  | Sulfohydrolase |  |
|  | Sulfate adynlyltransferase | cysN, cysC, sat, met3 |
| N metabolism | Nitrate/Nitrite transporter | NRT, nark, nrtP, nasA, nasB, cynA, cynB, nrtA, nrtB |
|  | Nitrate reductase | narG, narH, narI, narJ |
|  | Periplasmic nitrate reductase | napA, napB |
|  | Ferredoxin-nitrate reductase | narB |
|  | Nitrite reductase | nirK, nirS, nirB, nirD, nrfA, nrfH, NIT-6 |
|  | Nitronate monooxygenase |  |
|  | Nitroalkane oxidase |  |
|  | Nitric oxide reductase | norB, norC, CYP55 |
|  | Ferrodoxin-nitrite reductase | nirA |
|  | Hydroxylamine dehydrogenase | hao |
|  | Nitrous-oxide reductase (nitrous oxide reductase) | nosZ |
|  | Hydroxylamine reductase | hcp |
|  | Nitrogenase | nifD, nifK, nifH, anfG |
|  | Ammonia monooxygenase | amoA, amoB, amoC |
|  | Hydrazine oxidoreductase | hzs |

Supplementary Table 2. Contigs that contain *Nif*H, *Nif*D and *Nif*K gene sequences

| Sample | ContigID | GeneID | Putative gene | NR-best hit (by blastp) | | | |
| --- | --- | --- | --- | --- | --- | --- | --- |
|  |  |  |  | Phylum | Taxon | Accession number | % of identity |
| BE2011 | Contig52055 | 2023 | *Nif*H | Proteobacteria | *Thiorhodovibrio* sp. 970 | WP_009150597.1 | 88 |
|  |  | 2043 | *Nif*D | Proteobacteria | *Sideroxydans lithotrophicus* ES-1 | YP_003523507.1 | 85 |
|  |  | 2061 | *Nif*K | Proteobacteria | *Methylobacter tundripaludum* | WP_006892493.1 | 80 |
| BE2011 | Contig3324 | 577 | *Nif*H | Proteobacteria | *Ca*.*Accumulibacter* sp. BA-94 | YP_003523506.1 | 91 |
|  |  | 586 | *Nif*D | Proteobacteria | *Azoarcus* sp. BH72 | YP_932043.1 | 93 |
|  |  | 595 | *Nif*K | Proteobacteria | *Azoarcus* sp. BH72 | YP_932044.1 | 87 |
| BE2011 | Contig4829 | 19 | *Nif*H | Proteobacteria | *Ca*. *Accumulibacter phosphatis* clade IIA str. UW-1 | YP_003169604.1 | 90 |
|  |  | 29 | *Nif*D | Proteobacteria | *Sideroxydans lithotrophicus* ES-1 | YP_003523507.1 | 84 |
|  |  | 33 | *Nif*K | Proteobacteria | *Uliginosibacterium gangwonense* | WP_018607904.1 | 77 |
| BE2011 | Contig4971 | 1164 | *Nif*H | Proteobacteria | *Teredinibacter turnerae* | WP_019604939.1 | 89 |
|  |  | 1179 | *Nif*D | Proteobacteria | *Azoarcus* sp. BH72 | YP_932043.1 | 90 |
|  |  | 1191 | *Nif*K | Proteobacteria | *Pseudomonas stutzeri* A1501 | YP_001171865.1 | 81 |
| BE2011 | Contig8105 | 731 | *Nif*H | Proteobacteria | *Azoarcus* sp. BH72 | YP_932042.1 | 90 |
|  |  | 740 | *Nif*D | Proteobacteria | *Sideroxydans lithotrophicus* ES-1 | YP_003523507.1 | 84 |
|  |  | 746 | *Nif*K | Proteobacteria | *Uliginosibacterium gangwonense* | WP_018607904.1 | 78 |
| BE2011 | Contig9979 | 162 | *Nif*H | Firmicutes | *Ca*. *Desulforudis audaxviator* MP104C | YP_001716343.1 | 100 |
|  |  | 183 | *Nif*D | Firmicutes | *Ca*. *Desulforudis audaxviator* MP104C | YP_001716346.1 | 100 |
|  |  | 193 | *Nif*K | Firmicutes | *Ca*. *Desulforudis audaxviator* MP104C | YP_001716347.1 | 100 |
| BE2012 | Contig101417 | 178 | *Nif*H | Proteobacteria | *Thiorhodovibrio* sp. 970 | WP_009150597.1 | 88 |
|  |  | 198 | *Nif*D | Proteobacteria | *Sideroxydans lithotrophicus* ES-1 | YP_003523507.1 | 85 |
|  |  | 216 | *Nif*K | Proteobacteria | *Methylobacter tundripaludum* | WP_006892493.1 | 80 |
| BE2012 | Contig13449 | 187 | *Nif*H | Proteobacteria | *Ca*. *Accumulibacter phosphatis* clade IIA str. UW-1 | YP_003169604.1 | 90 |
|  |  | 197 | *Nif*D | Proteobacteria | *Sideroxydans lithotrophicus* ES-1 | YP_003523507.1 | 84 |
|  |  | 208 | *Nif*K | Proteobacteria | *Ca. Accumulibacter* sp. SK-02 | EXI80125.1 | 77 |
| BE2012 | Contig92166 | 120 | *Nif*H | Firmicutes | *Heliobacterium gestii* | BAE02721.1 | 85 |
|  |  | 144 | *Nif*D | Nitrospirae | *Thermodesulfovibrio yellowstonii* DSM 11347 | YP_002249507.1 | 72 |
|  |  | 175 | *Nif*K | Cyanobacteria | *Leptolyngbya boryana* | WP_017289031.1 | 64 |
| BE2012 | Contig8052 | 162 | *Nif*H | Firmicutes | *Ca. Desulforudis audaxviator* MP104C | YP_001716343.1 | 100 |
|  |  | 183 | *Nif*D | Firmicutes | *Ca. Desulforudis audaxviator* MP104C | YP_001716346.1 | 100 |
|  |  | 193 | *Nif*K | Firmicutes | *Ca. Desulforudis audaxviator* MP104C | YP_001716347.1 | 100 |
| DR5 | Contig3575 | 120 | *Nif*H | Firmicutes | *Heliobacterium gestii* | BAD80880.1 | 81 |
|  |  | 143 | *Nif*D | Nitrospirae | *Thermodesulfovibrio yellowstonii* DSM 11347 | YP_002249507.1 | 71 |
|  |  | 167 | *Nif*K | Nitrospirae | *Thermodesulfovibrio yellowstonii* DSM 11347 | YP_002249506.1 | 66 |
| FI88 | Contig10420 | 816 | *Nif*H | Proteobacteria | *Desulfovibrio africanus* | WP_005986594.1 | 88 |
|  |  | 847 | *Nif*D | Proteobacteria | *Desulfospira joergensenii* | WP_022667734.1 | 77 |
|  |  | 863 | *Nif*K | Proteobacteria | *Desulfobacca acetoxidans* DSM 11109 | YP_004369426.1 | 74 |
| FI88 | Contig25282 | 397 | *Nif*H | Proteobacteria | *Teredinibacter turnerae* | WP_019604939.1 | 89 |
|  |  | 411 | *Nif*D | Proteobacteria | *Thiothrix nivea* | WP_002710380.1 | 87 |
|  |  | 423 | *Nif*K | Proteobacteria | *Beggiatoa alba* | WP_002685512.1 | 81 |
| FI88 | Contig3844 | 1094 | *Nif*H | Proteobacteria | *Desulfobacca acetoxidans* DSM 11109 | YP_004369422.1 | 87 |
|  |  | 1129 | *Nif*D | Proteobacteria | *Desulfatibacillum alkenivorans* AK-01 | YP_002430685.1 | 79 |
|  |  | 1152 | *Nif*K | Proteobacteria | Uncultured *Desulfobacterium* sp. | CBX26826.1 | 77 |
| FI88 | Contig44259 | 1474 | *Nif*H | Proteobacteria | *Teredinibacter turnerae* | WP_019604939.1 | 89 |
|  |  | 1488 | *Nif*D | Proteobacteria | *Thiothrix nivea* | WP_002710380.1 | 87 |
|  |  | 1500 | *Nif*K | Proteobacteria | *Beggiatoa alba* | WP_002685512.1 | 81 |
| FI88 | Contig6691 | 968 | *Nif*H | Proteobacteria | *Sideroxydans lithotrophicus* ES-1 | YP_003523506.1 | 94 |
|  |  | 983 | *Nif*D | Proteobacteria | *Azoarcus* sp. BH72 | YP_932043.1 | 89 |
|  |  | 1001 | *Nif*K | Proteobacteria | *Sideroxydans lithotrophicus* ES-1 | YP_003523508.1 | 85 |
| TT109 | Contig6944 | 509 | *Nif*H | Proteobacteria | *Marinobacter* sp. ES-1 | WP_022990315.1 | 90 |
|  |  | 523 | *Nif*D | Proteobacteria | *Ca. Accumulibacter phosphatis* clade IIA str. UW-1 | YP_003169605.1 | 84 |
|  |  | 536 | *Nif*K | Proteobacteria | *Uliginosibacterium gangwonense* | WP_018607904.1 | 78 |
| TT109 | Contig85435 | 915 | *Nif*H | Nitrospirae | *Thermodesulfovibrio yellowstonii* DSM 11347 | YP_002249508.1 | 92 |
|  |  | 935 | *Nif*D | Nitrospirae | *Thermodesulfovibrio yellowstonii* DSM 11347 | YP_002249507.1 | 95 |
|  |  | 955 | *Nif*K | Nitrospirae | *Thermodesulfovibrio yellowstonii* DSM 11347 | YP_002249506.1 | 96 |
| TT109 | Contig5544 | 2113 | *Nif*H | Proteobacteria | *Bradyrhizobium* sp. S23321 | YP_005451908.1 | 93 |
|  |  | 2127 | *Nif*D | Proteobacteria | *Methylococcus capsulatus* str. Bath | YP_112765.1 | 95 |
|  |  | 2141 | *Nif*K | Proteobacteria | *Methylococcus capsulatus* str. Bath | YP_112766.1 | 86 |
| TT109 | Contig7492 | 4 | *Nif*H | Proteobacteria | *Ectothiorhodospira* sp. PHS-1 | WP_008932809.1 | 86 |
|  |  | 14 | *Nif*D | Proteobacteria | *Ca. Accumulibacter phosphatis* clade IIA str. UW-1 | YP_003169605.1 | 84 |
|  |  | 23 | *Nif*K | Proteobacteria | *Uliginosibacterium gangwonense* | WP_018607904.1 | 80 |

Supplementary Figure 1 (next page). Genus-level taxonomic distribution of 8 common functional genes detected in the subsurface metagenomes. N denotes the total number of gene sequences detected in the metagenomes. The taxonomic classification of these sequences was assigned based on the lineage of their NR-best hit. The common functional genes are denoted by letters: a: *Nar*V gene; b: NPD gene; c: PAPS reductase gene; d: *Nif*H gene; e: *Nif*D gene; f: *Nif*K gene; g: *Nif*E gene; h: *Nif*N gene. Sequence counts were also overlain with scaled color intensity for visual effect (blue for low values and red for high values). The last row gives the number of phyla represented by each common functional gene. This figure is best viewed with at least 200% magnification.
